# Long-term survival after conversion surgery for an esophageal neuroendocrine carcinoma: a case report

**DOI:** 10.1186/s44215-024-00155-5

**Published:** 2024-05-10

**Authors:** Takahiro Enjoji, Shinichiro Kobayashi, Kohei Hayashi, Hanako Tetsuo, Ryo Matsumoto, Yasuhiro Maruya, Tomonori Araki, Takuya Honda, Yuko Akazawa, Kengo Kanetaka, Kazuhiko Nakao, Susumu Eguchi

**Affiliations:** 1 Department of Surgery, Nagasaki University Graduate School of Biomedical Sciences, Nagasaki, Japan; 2 Department of Gastroenterology and Hepatology, Graduate School of Biomedical Sciences, Nagasaki University, Nagasaki, Japan; 3 Department of Histology and Biomedical Sciences, Graduate School of Biomedical Sciences, Nagasaki University, Nagagasaki, Japan; 4 Department of Surgery, juzenkai hospital, Nagagasaki, Japan; 5 Present Address: Department of Surgery, Nagasaki Harbor Medical Center, Nagasaki, Japan; 6 Hospital Directer, Sasebo city general hospital, Nagagasaki, Japan; 7 Department of Surgery, Yamaguchi Prefectural Grand Medical Center, Hofu, Yamaguchi, Japan

**Keywords:** Conversion surgery, Esophagus, Neuroendocrine carcinoma

## Abstract

**Background:**

Esophageal neuroendocrine carcinomas (NECs) are extremely rare. Published information on their clinical features, pathological findings, and prognosis is lacking. The optimal treatment for esophageal NECs has not yet been determined because they appear extremely malignant histologically and have a poor prognosis. We here report on a patient with an esophageal NEC who was successfully treated with multidisciplinary therapies.

**Case presentation:**

The patient, a 47-year-old man, was admitted to our hospital with dysphagia and weight loss and an ECOG performance status of 3–4. Upper endoscopy showed a large circumferential neoplasm at the esophagogastric junction. Computed tomography showed lymph node metastases around the left gastric artery. Echocardiography raised a suspicion of invasion of the left atrium. Histopathological examination of biopsy tissue revealed diffuse proliferation of small atypical cells resembling naked nuclei with extensive necrosis and degeneration. The cells showed positive staining for CD56, chromogranin A, and synaptophysin on immunohistochemical analysis. Ki67 was positive in over 90% of cells. He was diagnosed with an esophageal NEC that had infiltrated the left atrium; his cancer was therefore inoperable. Because of his poor overall condition, palliative radiotherapy (30 Gy in 15 fractions) was administered, accompanied by nutritional management. This was followed by four courses of chemotherapy with carboplatin plus etoposide, after which the primary tumor and locoregional lymph node metastases were found to have shrunk. Thoracoscopic- and laparoscopic-assisted McKeown esophagectomy were performed. The postoperative pathological diagnosis was NEC pT3pN0M0 Stage II. The patient had a good postoperative course and received two courses of postoperative adjuvant chemotherapy (carboplatin plus etoposide). Currently, 3 years after commencing treatment, there is no evidence of recurrence.

**Conclusions:**

As mentioned above, there is no established treatment regime for esophageal NEC, and the benefits of conversion surgery are unknown. Our patient achieved long-term recurrence-free survival after radiation therapy, chemotherapy, and surgery for an esophageal NEC with left atrial invasion and multiple lymph node metastases. Conversion surgery for esophageal NECs that respond to chemotherapy may contribute to long-term survival.

## Background

Neuroendocrine carcinomas (NECs) are rare, the reported incidence being 0.4–2% of all esophageal malignancies [1].

Unlike typical esophageal cancers, esophageal NECs require a distinct treatment approach. Because they have a high rate of systemic recurrence and generally poor prognosis, treatment guidelines advocate a multidisciplinary strategy encompassing surgery, chemotherapy, and radiation therapy [2]. When resection is not feasible at complex anatomic sites such as the esophagus, a combination of radiation therapy and chemotherapy is advised. Currently, no consensus on the role of conversion surgery in treating esophageal NECs is available [2]. We here describe a patient with an esophageal NEC who achieved long-term survival following conversion surgery after initial radiation therapy and chemotherapy.

## Case presentation

A 47-year-old man with a significant smoking history (30–40 cigarettes daily for 26 years) and history of pneumothorax and appendectomy presented with severe dysphagia and a rapid weight loss of over 5 kg within a week. He was initially admitted to another hospital, but a swift decline in his general condition, marked by prominent emaciation and a performance status of 3–4, prompted his referral to our facility.

**Fig. 1:**
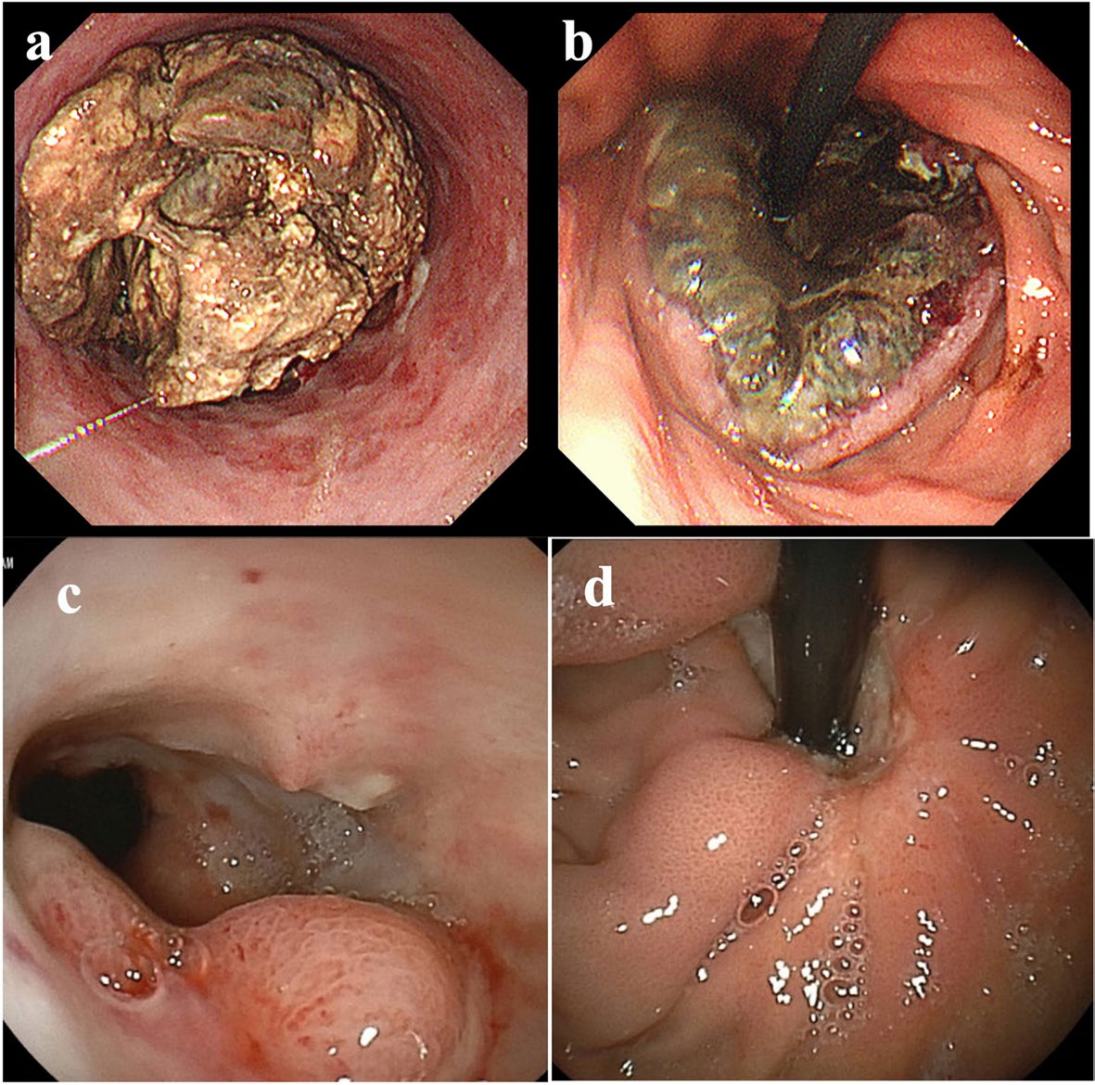
Upper endoscopy findings. **a** Initial upper endoscopy revealed a circumferential type 3 lesion at the esophagogastric junction with approximately 10 cm of esophageal invasion. **b** Upper endoscopy after two courses of chemotherapy showing tumor shrinkage and improvement in obstruction

**Fig. 2:**
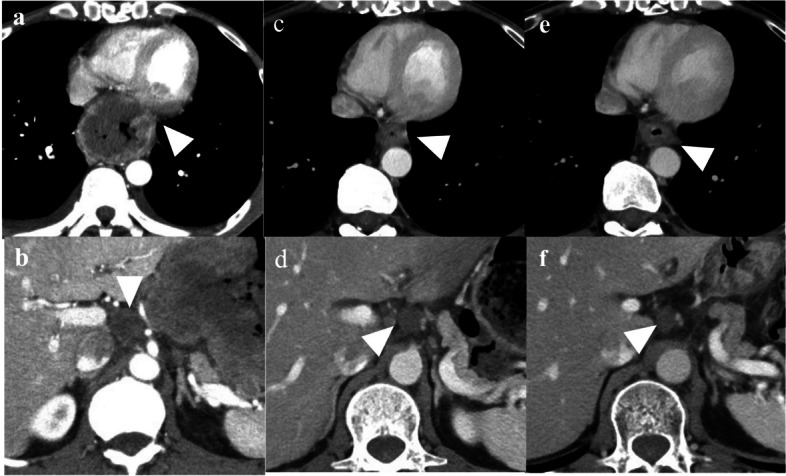
Enhanced CT findings. **a** CT images showing a large mass at the esophageal junction. Tumor invasion into the left atrium was suspected (white arrow head). **b** Intra-abdominal lymph node metastases were identified (white arrow head). **c** Enhanced CT images after two courses of chemotherapy showing that the primary tumor has decreased in size (white arrow head). **d** The lymph node metastases remain shrunk (white arrow head). **e** Enhanced CT images after four courses of chemotherapy showing that the primary tumor has enlarged slightly (white arrow head). **f** The lymph node metastases have also decreased in size (white arrow head)

**Fig. 3:**
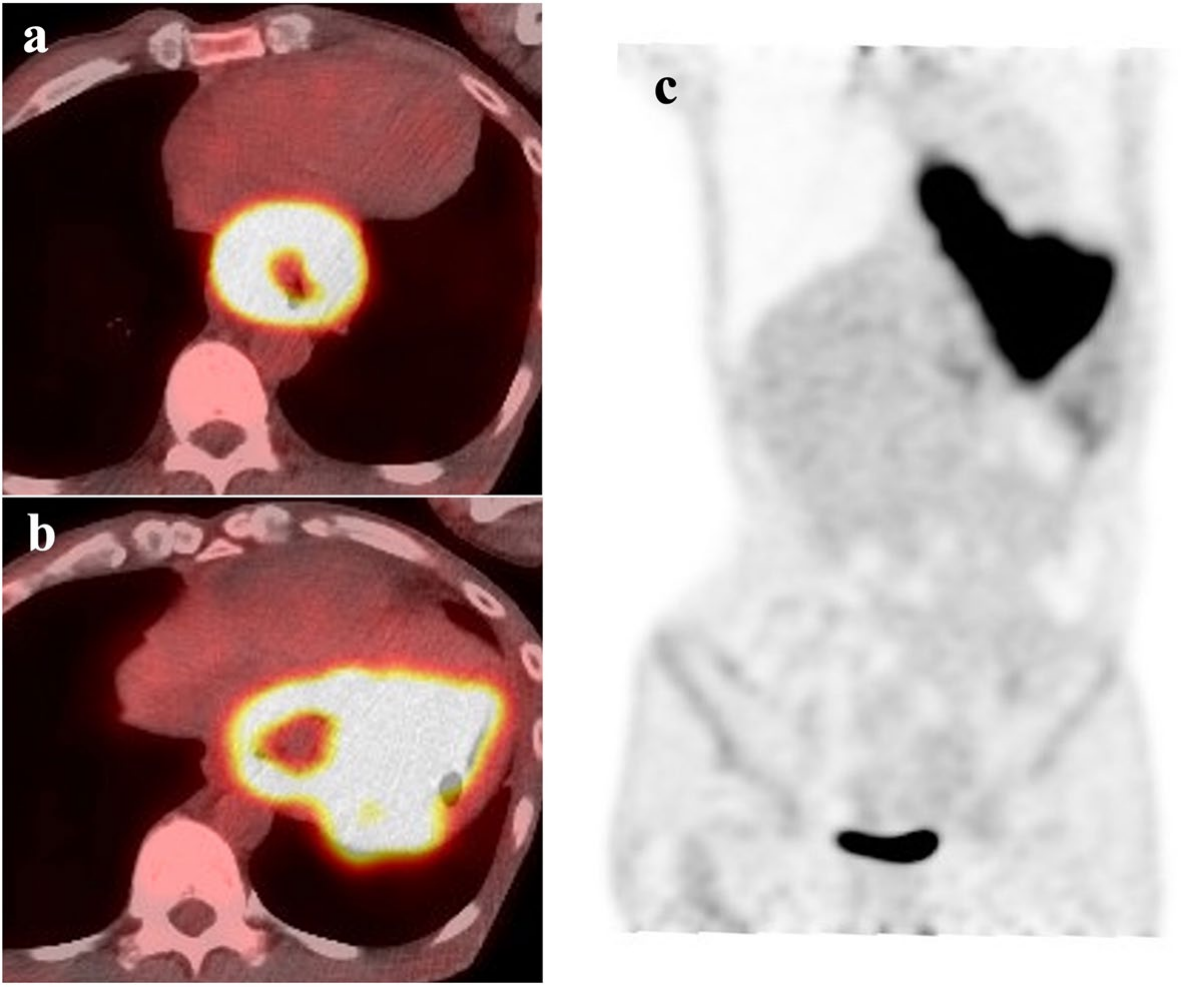
PET-CT findings. **a** and **b** Initial PET-CT showed hypermetabolism of esophageal tumor (SUVmax = 13.355). **c** No metastases to distant organs were detected

**Fig. 4:**
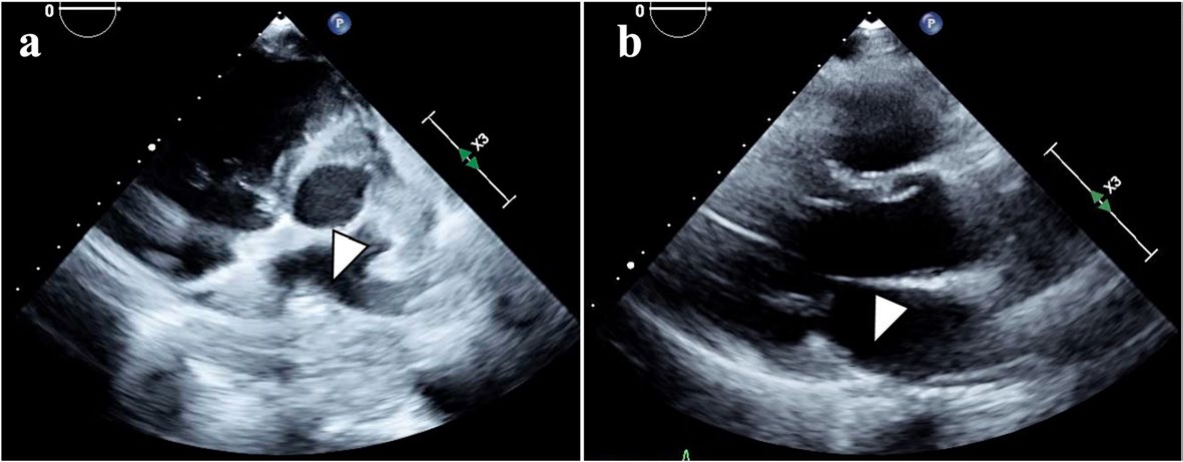
Echocardiography findings. **a** Initial echocardiogram showed hyperintense tumor invasion into the left atrium (white arrow head). **b** Echocardiography after four courses of chemotherapy showing no evidence of tumor invading the left atrium (white arrow head)

**Fig. 5:**
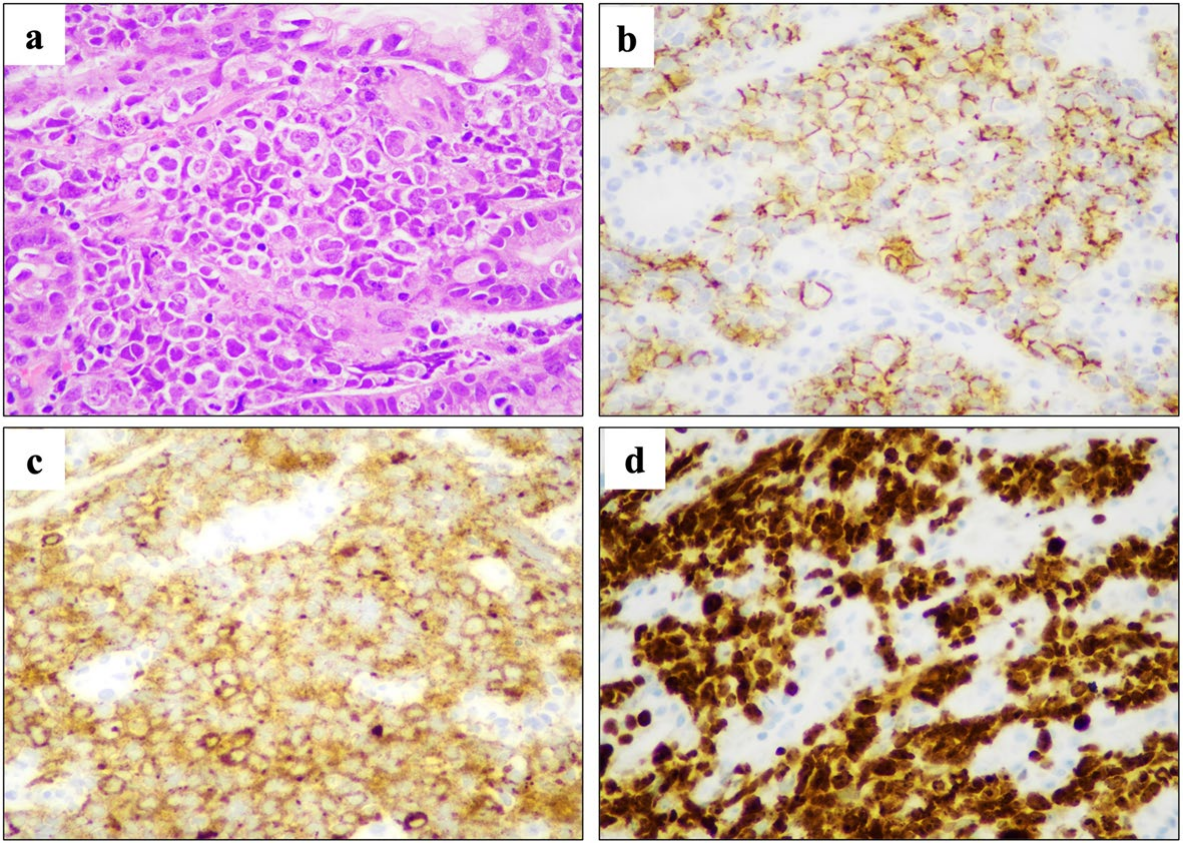
Histological and immunohistochemical findings. **a** Hematoxylin and eosin staining, along with histochemical staining (**b**)~(**d**), is shown for the biopsy specimen obtained from the gastric side of the tumor. The tumor exhibited positive staining for CD56 (**b**) and synaptophysin (**c**). More than 90% of the tumor cells were positive for Ki67 (**d**). Original magnification, × 40

Upper gastrointestinal endoscopy revealed a circumferential Borrmann type 3 tumor at the esophagogastric junction involving approximately 100 mm of the esophagus (Fig. 1a, b). Computed tomography (CT) showed a 100 × 110 mm tumor at the esophagogastric junction and possible invasion of the left atrium. Notably, the scan also showed enlarged lymph nodes around the left gastric artery, indicative of lymph node metastases (Fig. 2a, b). Positron emission tomography/computed tomography (PET/CT) revealed a hypermetabolic esophageal tumor and regional lymph nodes with no evidence of distant metastasis (Fig. 3a, b, c). Echocardiography also revealed possible tumor invasion of the left atrium (Fig. 4a). Histopathological examination of biopsy tissue revealed diffuse proliferation of small atypical cells resembling naked nuclei with extensive necrosis and degeneration. Immunohistochemical staining results were as follows: CD56 (+), chromogranin A (+), synaptophysin (+), and Ki-67 positivity > 90%, confirming the diagnosis of an esophageal NEC (Fig. 5).

**Fig. 6:**
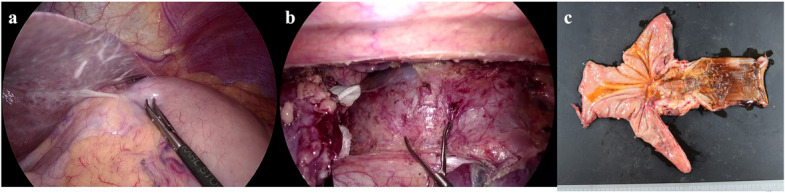
Surgical findings and excised specimens. **a** Staging laparoscopy findings. The abdominal lesion was judged to be resectable because the lesion had shrunk with chemotherapy. **b** Thoracoscopy findings. It proved possible to separate the esophageal lesion from the pericardial sac and there was no residual tumor. **c** Photograph of the resected esophagus

We deemed the tumor inoperable because of the infiltration of the left atrium. Owing to the patient’s poor overall condition, palliative radiotherapy (30 Gy in 15 fractions) was administered, accompanied by nutritional management. This achieved improvement in his ability to eat and overall performance status, making him fit to undergo chemotherapy with carboplatin and etoposide. A repeat CT scan following two courses of chemotherapy showed that both the primary tumor and lymph node metastases had shrunk dramatically, and there was no longer any evidence suggestive of tumor invasion of the left atrium (Fig. 2c, d). A cardiac echocardiogram also found no infiltration of the tumor into the left atrium (Fig. 4b). Post-chemotherapy upper endoscopy also demonstrated tumor shrinkage and improved esophageal patency (Fig. 1c, d). However, after four courses of chemotherapy, despite the lymph node metastases continuing to shrink, the primary tumor had enlarged slightly, and the patient’s dysphagia had progressively worsened (Fig. 2e, f). We therefore performed a thoracoscopic esophagectomy and two-region thoracoabdominal dissection. Intraoperatively, there was no tumor invasion of the left atrium (Fig. 6a, b). We performed lymph node dissection for the lower mediastinal lymph nodes (LN108, 110, 112ao, 112pul) and abdominal lymph nodes (LN1, 2, 3, 7, 8a, 9, 11, 16a2), with a total of 5 lower mediastinal lymph nodes and 34 abdominal lymph nodes dissected. The postoperative pathological diagnosis was [neuroendocrine carcinoma E = G type3 crt-pT3pN0M0 crt-f StageII] [3, 4]. The findings indicated NEC, large cell type, with no coexistence of squamous cell carcinoma or adenocarcinoma. Necrosis was observed in the dissected lymph nodes LN7 and LN8, but viable cancer cells were not identified. And the therapeutic effect was grade 1a (Fig. 6c) [5].

The patient’s postoperative course was uneventful. Due to the satisfactory control of metastatic lesions with preoperative carboplatin and etoposide, two courses of adjuvant chemotherapy were administered using the same regimen. Remarkably, he remains free of recurrence 3 years after the surgery.

## Discussion

The recommended treatment for gastrointestinal NECs is surgery and/or adjuvant therapy for local disease and chemoradiotherapy for locoregional disease [2, 6]. However, in the case of esophageal NECs, the high morbidity associated with esophagectomy necessitates a more nuanced approach to determining eligibility for surgery.

The use of platinum-based chemotherapy, often in combination with etoposide, reflects the standard treatment for small cell lung cancer, highlighting the cross-application of oncological therapies [6]. The roles of alternative regimens, such as cisplatin/irinotecan, also merit consideration given their efficacy against related cancers.

According to a recent study, median survival after radical esophagectomy and regional lymphadenectomy for esophageal NECs ranges from 10.1 to 28.5 months and median survival after chemoradiation therapy alone from 8.0 to 16.1 months [1]. Survival outcomes with chemoradiotherapy alone are typically poorer, underscoring the importance of seriously considering surgical intervention. On the other hand, in patients with stages III and IV esophageal NEC, there have been reports suggesting that the prognosis of patients who received chemoradiotherapy was better than those who underwent surgery plus chemotherapy. However, it is noted that in surgical treatment, the lack of preoperative chemotherapy and the immunosuppressive effects due to surgical invasion may impact the prognosis. Therefore, it is emphasized that for advanced esophageal NEC, it is important to appropriately combine local treatment (surgery) with systemic treatment [7].

Conversion surgery, defined as surgery following successful downstaging of initially unresectable tumors via chemotherapy, is a relatively novel concept in the realm of esophageal NECs. Although few published reports specifically address conversion surgery for esophageal NEC, data from trials in other unresectable esophageal cancers suggest that outcomes might be promising [8, 9]. A Phase II trial on conversion surgery for initially unresectable esophageal cancer after treatment with docetaxel, cisplatin, and 5-fluorouracil has shown favorable results with an esophagectomy rate of 42% and R0 resection rate of 95% [10]. It is anticipated that an ongoing Phase III JCOG1510 trial, which compares radical chemoradiotherapy with conversion surgery post-induction with docetaxel, cisplatin, and 5-fluorouracil for unresectable esophageal cancer, will provide valuable insights that will potentially influence future treatment guidelines (https://pubmed.ncbi.nlm.nih.gov/31411696/). However, to the best of our knowledge, no cases of conversion surgery for esophageal NEC have been reported.

Determination of eligibility for surgery and the extent of resection in conversion surgery for esophageal NECs are complex. Complete resection of gastric adenocarcinoma in conversion surgery reportedly has favorable prognoses [11–13], underscoring the potential benefit of a similar approach to esophageal NECs. The optimal extent of lymph node dissection during conversion surgery for esophageal NEC remains unclear. Studies targeting T2–T4 EGJ cancer have reported that when esophageal involvement exceeds 2 cm, lymph nodes with a metastasis rate of over 10% are found in the abdomen at LN 1, 2, 3, 7, 9, and 11p and in the lower mediastinum at LN 110 [3]. In this case, dissection was only performed for the lymph nodes with metastasis and those with a metastasis rate exceeding 10%. Administration of early adjuvant chemotherapy is also critical in improving postoperative outcomes for advanced esophageal NECs. Therefore, preventive lymph node dissection should not be performed with esophagectomy; instead, the least aggressive surgical procedure that will achieve surgical complete resection while minimizing the risk of complications should be performed.

## Conclusions

This case of esophageal NEC highlights the potential of conversion surgery to achieve long-term survival in selected patients. It underscores the need for individualized treatment approaches, careful selections of multiple treatment modalities, and the importance of timing of conversion surgery in managing this challenging malignancy.

## Data Availability

Not applicable.

## Abbreviations

CT: Computed tomography
NEC: Neuroendocrine carcinoma
PET/CT: Positron emission tomography/computed tomography
